# LncRNA H19 induced by helicobacter pylori infection promotes gastric cancer cell growth via enhancing NF-κB-induced inflammation

**DOI:** 10.1186/s12950-019-0226-y

**Published:** 2019-11-26

**Authors:** Yifeng Zhang, Jin Yan, Chao Li, Xiaoyong Wang, Yu Dong, Xiaoran Shen, Ximei Zhang

**Affiliations:** 1 Department of Gastroenterology, Nanjing First Hospital, Nanjing Medical University, No.68 Changle Road, Qinhuai District, Nanjing, 210000 Jiangsu China; 2 Department of Gastroenterology, The First Affiliated Hospital of Nanjing Medical University, Nanjing, 210000 Jiangsu China; 3 Department of Gastroenterology, The No. 2 Hospital of Changzhou, Changzhou, 211166 Jiangsu China; 4 Department of Gastroenterology, The No. 1 People’s Hospital of Nantong, Nantong, 226001 Jiangsu China

**Keywords:** H19, *H. pylori*, Gastric cancer, NF-κB signaling pathway

## Abstract

**Background:**

The aim of this study was to investigate the role of long non-coding RNA (lncRNA) H19 in gastric cancer (GC) with Helicobacter pylori (*H. pylori*).

**Methods:**

H19 expression in peripheral blood from *H. pylori*+/− GC patients and healthy donors (control) as well as in GC tissues and cells were detected by qRT-PCR. Cell proliferation was evaluated by CCK-8 assay. Cell migration and invasion were evaluated by Transwell assay. The levels of pro-inflammatory cytokines were determined by ELISA. The protein levels of IκBα, p-IκBα and p65 were determined by western blotting.

**Results:**

H19 expression was upregulated in *H. pylori*-infected GC tissues and cells. Furthermore, *H. pylori* promoted GC cell viability, migration, invasion and inflammatory response. Moreover, H19 overexpression promoted the proliferation, migration and invasion of *H. pylori*-infected GC cells via enhancing NF-κB-induced inflammation.

**Conclusions:**

LncRNA H19 promotes *H. pylori*-induced GC cell growth via enhancing NF-κB-induced inflammation.

## Background

Gastric cancer (GC) is the fifth most common cancer, imposing a global cancer burden [1]. Although the mortality rate of GC has declined in recent years [2], many patients are diagnosed at an advanced stage with lymphatic or distant metastasis without specific symptoms. The 5-year survival rate of stage III GC is only approximately 15%. However, available therapeutic approaches for advanced GC are limited [3–5]. Therefore, it is of great importance to discover new molecular mechanisms and therapeutic targets that control the severity of GC and present predictive value for prognosis. Helicobacter pylori (*H. pylori*, HP) is perhaps one of the most common human infectious agents worldwide [6]. At present, HP is considered to be the most common etiologic agent for infection-related cancers, which represent 5.5% of the global cancer burden [7]. Despite a close causal link between HP infection and the development of gastric malignancies [8], the precise mechanisms involved in this process are still obscure. HP has been shown to induce gastric mucosa epithelial cells and GC cells to release cytokines including IL-1β, IL-6, IL-8 and TNF-α [9, 10]. Emerging evidence indicates that HP induces IL-8 secretion in gastric epithelial cells via classical activation pathway of NF-κB signaling, which has been identified as regulating several sporadic and inflammation-associated gastrointestinal tract malignancies [11, 12]. It has been shown that HP can induce the catalytic activity of the IκB kinases (IKKα and IKKβ) and promote IκB degradation in GC [13, 14]. Long non-coding RNAs (lncRNAs) are generally defined as RNA transcripts longer than 200 nucleotides without protein-coding function. An increasing number of non-coding RNAs have been found to play critical roles in cancer development and metastasis [15]. LncRNA H19 was discovered in 1991 by Bartolomei and shown to lack a common open reading frame in the RNA or an encoded protein [16, 17]. H19 has emerged as a vital regulatory molecule in tumorigenesis [18]. Our previous work showed that H19 was increased in GC cell lines and tissues, and H19 overexpression promoted gastric cell proliferation and inhibited cell apoptosis, whereas H19 knockdown yielded the opposite results [19]. Importantly, H19 expression was upregulated in the serum of patients with GC with HP infection [20]. However, the role of H19 in GC with HP infection remains unclear. In this study, we investigated the role of H19 in regulating proliferation, migration and invasion of HP-induced GC cells. Furthermore, we elucidated whether the underlying mechanism was associated with its regulation of NF-κB signaling pathway.

## Materials and methods

### Human tissue samples

Paired GC tissue samples and corresponding adjacent noncancerous gastric samples of patients were collected from Nanjing First Hospital, Nanjing Medical University (Nanjing, China). All samples were confirmed as GC by pathological analysis and none of the patients had received chemotherapy or radiotherapy before surgical resection. Informed consent was obtained from all patients and this study was approved by the Ethical Committee of the Nanjing First Hospital, Nanjing Medical University.

### Cell lines and cell culture

Human GC cell line SGC-7901 and normal gastric epithelial cell line GES-1 were purchased from the Shanghai Institute of Cell Biology (Shanghai, China). Cells were cultured in RPMI-1640 (Invitrogen, Carlsbad, CA, USA) supplemented with 10% fetal bovine serum (FBS; Invitrogen, Carlsbad, CA, USA) and 1% antibiotics. Cells were incubated in a humidified incubator in an atmosphere of 5% CO_2_ at 37 °C.

### Cell transfection

To overexpress H19, the full-length sequences of H19 were subcloned into pcDNA3.1 vector (Invitrogen) referred as pcDNA3.1-H19, with an empty pcDNA3.1 vector used as a control. To silence H19, a siRNA targeting H19 (si-H19), and control scramble siRNA (si-ctrl) were synthesized by GenePharma (Shanghai, China). The siRNA sequences for lncRNA H19 was as follows: si-H19, 5′-CCAACAUCAAAGACACCAUdTdT-3′ and si-ctrl, 5′-AUUUCUUUCAUGUUGUGGGTT-3′. Transfection was performed using Lipofectamine 2000 (Invitrogen) according to the manufacturer’s protocol. Forty-eight hours after transfection, transfected cells were collected and used in further experiments.

### HP strains and infection model

The HP strain 11637 (obtained from ATCC) were grown on brain-heart infusion plates containing 10% rabbit blood at 37 °C under microaerophilic conditions (5% O_2_, 10% CO_2_, and 85% N_2_). HP was washed from the culture plates with sterile PBS. The suspended HP was centrifuged at 2500×g for 5 min and re-suspended in RPMI-1640 medium without antibiotics. The amount of bacteria was determined by measuring optical density at 600 nm (1 OD600 = 1 × 109 CFU/ml). RPMI-1640 medium alone served as a blank control. Cultured cells were seeded on plates and grown to 80% confluence. Then, HP was added to cells at a bacteria-to-cell ratio of 1:1, 10:1, or 100:1 (multiplicity of infection (MOI) = 0, 1, 10, 100).

### Quantitative real-time PCR (qRT-PCR)

Total RNA was extracted from GC tissues, SGC-7901 and GES-1 cells using TRIzol reagent (Invitrogen, USA) following the manufacturer’s protocol. The cDNA was synthesized using a reverse transcription kit. The qRT-PCR was performed with a MiniOpticon real-time PCR device. Data were normalized to the reference gene GAPDH for each cDNA sample. The primers used in this study were as follows: H19 forward: ACCACTGCACTACCTGACTC; reverse: CCGCAGGGGGTGGCCATGAA. GAPDH forward: GAGTCAACGGATTTGGTCGT; reverse: GACAAGCTTCCCGTTCTCAG. The expression of H19 was calculated with the 2^-△△Ct^ method and normalized to GAPDH.

### Cell proliferation assay

The proliferation of SGC-7901 cells following transfection was measured using the Cell Counting Kit-8 (CCK-8; Dojindo Laboratories, Kumamoto, Japan). Briefly, after transfection, approximately 2 × 10^3^ cells were seeded in each well of a 96-well plate, and 10 μl of CCK-8 solution (Dojindo, Kumamoto, Japan) was added to 90 μl of the culture medium at the indicated time at 37 °C in 5% CO_2_ according to the manufacturers’ instruction. Finally, the ultraviolet absorbance at 450 nm of each well was measured using a microplate reader (Bio-Rad, USA).

### Cell migration and invasion assay

For the transwell migration assay, cells were suspended in 100 μl of serum-free medium and then plated in the upper chamber of each insert (8-μm pore size, Corning, USA). Lower chambers of the inserts were filled with DMEM medium with 10% FBS. After 24 h of incubation, cells that migrated to the lower surface of the insert were fixed, stained with 0.1% crystal violet, and counted and averaged from five randomly chosen fields for each well under a light microscope. Each experiment was performed in triplicate wells and repeated three times. Cell invasion assay was performed the same way as the cell migration assay as described above, except that the upper chambers of 24-well Transwell plates were precoated with a Matrigel-coated membrane (BD Bioscience, San Jose, USA).

### Enzyme-linked immunosorbent assay (ELISA)

The levels of TNF-α, IL-1β, IL-6 and IL-8 in SGC-7901 cells were measured using their commercial ELISA kits (Abcam, Cambridge, UK) according to the manufacturer’s instructions.

### Western blotting

Proteins were extracted with RIPA buffer. The nuclear extracts were prepared using a NE-PER Nuclear and Cytoplasmic Extraction Reagents (Thermo Scientific, Rockford, USA) according to the manufacturer’s instructions. The protein concentration was determined using a protein assay kit (Bio-Rad). Approximately 30 μg of protein from each sample was separated on a 10% SDS-polyacrylamide gel and transferred to polyvinylidene difluoride membranes. After being blocked with 5% skim milk, the membranes were incubated with IκBα antibody (Abcam Cambridge, UK), p-IκBα antibody (Abcam Cambridge, UK) and p65 antibody (Abcam Cambridge, UK) overnight at 4 °C, followed by incubation with the corresponding secondary antibodies for 1 h at room temperature. Proteins were detected using Super ECL Plus Detection Reagent (Thermo Fisher Scientific, Carlsbad, CA, USA). β-actin was used as an endogenous control for total and cytosolic extracts. As for NF-κB nuclear p65, Lamin B served as the nuclear loading control.

### Statistics

The data are presented as mean ± standard deviation (SD) from three independent experiments. Differences between means were determined by Student’s t-test in two groups, or ANOVA followed by Tukey post-hoc test if more than two groups. *P* < 0.05 was considered statistically significant.

## Results

### H19 was upregulated in HP-infected GC tissues and cells

**Fig. 1:**
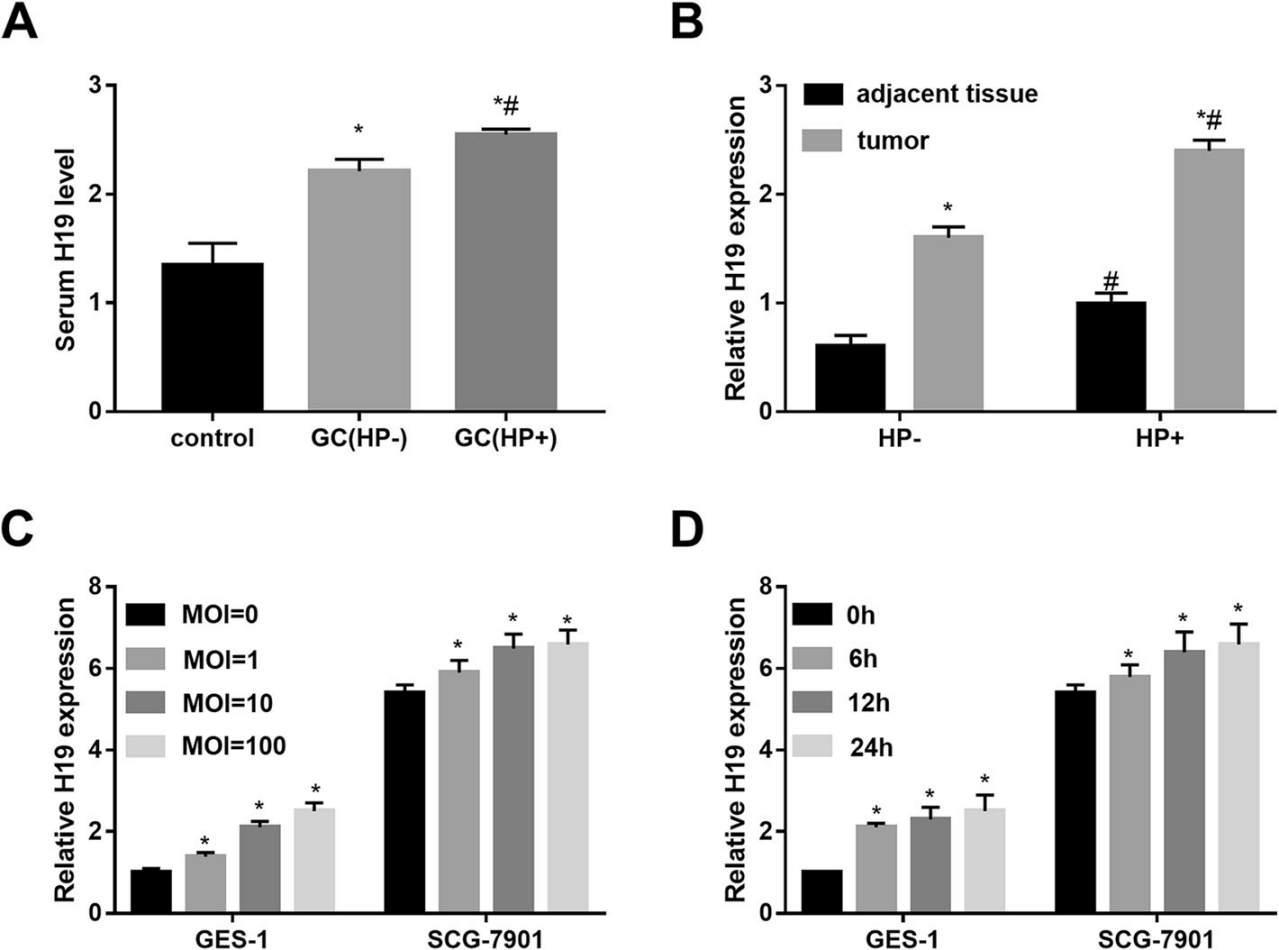
H19 was upregulated in HP-infected GC tissues and cells. **a**: H19 expression in sera from HP +/− GC patients and healthy donors (control) was detected by qRT-PCR. **b**: H19 expression in gastric carcinoma and adjacent normal tissues from HP +/− GC patients was detected by qRT-PCR. *N* = 30 for each group. **c**: H19 expression in SGC-7901 cells and gastric mucosa epithelial GES-1 cells following 24 h of HP infection at MOI (=0, 1, 10, 100) was detected by qRT-PCR. D: H19 expression in SGC-7901 and GES-1 cells following HP infection at MOI = 100 for 0, 6, 12, 24 h was detected by qRT-PCR. Each experiment was repeated three times. *p < 0.05 vs. control or adjacent tissue or MOI =0 or 0 h; #*p* < 0.05 vs. HP-

qRT-PCR analysis showed that serum H19 expression was significantly higher in HP +/− GC patients relative to control donors, and higher H19 expression was also observed in HP+ group compared with HP- group (Fig. 1a). Furthermore, H19 expression was significantly upregulated in GC tumors from both in HP+ and HP- patients relative to adjacent tissues, and H19 expression was also prominently upregulated in HP+ tissues compared with HP- group (Fig. 1b). We further infected SGC-7901 GC cells and gastric mucosa epithelial GES-1 cells with HP at MOI (0, 1, 10, 100). qRT-PCR analysis revealed that H19 expression was significantly higher in the MOI = 1, 10, 100 group than in that in the MOI = 0 group (Fig. 1c). Furthermore, HP infection at MOI = 100 increased H19 expression in a time-dependent manner (Fig. 1d). These data indicated that H19 expression was upregulated in GC tissues and cells with HP infection.

### HP infection promoted GC cell viability, migration, invasion and inflammatory response

**Fig. 2:**
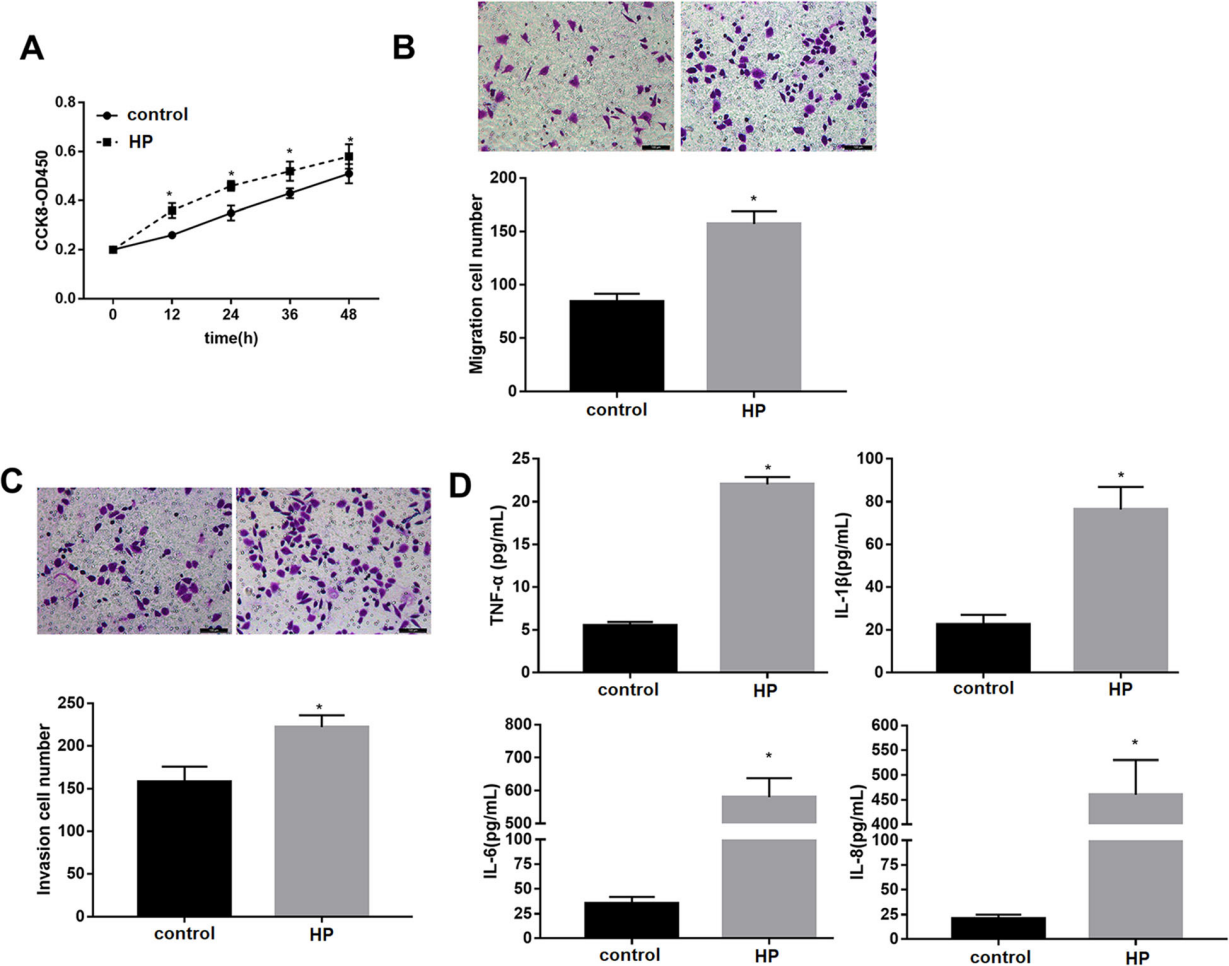
The effect of HP infection on GC cell behaviors. SGC-7901 cells were infected with HP at MOI = 100 for 24 h. **a**: Cell viability was analyzed using a CCK-8 kit. **b**, **c**: Cell migration and invasion were analyzed using transwell assays. **d**: The levels of pro-inflammatory cytokines were determined by ELISA. Each experiment was repeated three times. *p < 0.05 vs. control

Next, we investigated the effect of HP infection on GC cell behavior. Data revealed that HP infection significantly promoted cell viability within 48 h (Fig. 2a) as well as cell migration (Fig. 2b) and invasion (Fig. 2c) in SGC-7901 cells. In addition, HP infection (MOI = 100) markedly elevated levels of pro-inflammatory cytokines including TNF-α, IL-1β, IL-6 and IL-8 (Fig. 2d) in SGC-7901 cells. These observations indicated HP promoted GC cell viability, migration, invasion and inflammatory response.

### H19 promoted HP-induced GC cell proliferation, cell migration and invasion

**Fig. 3:**
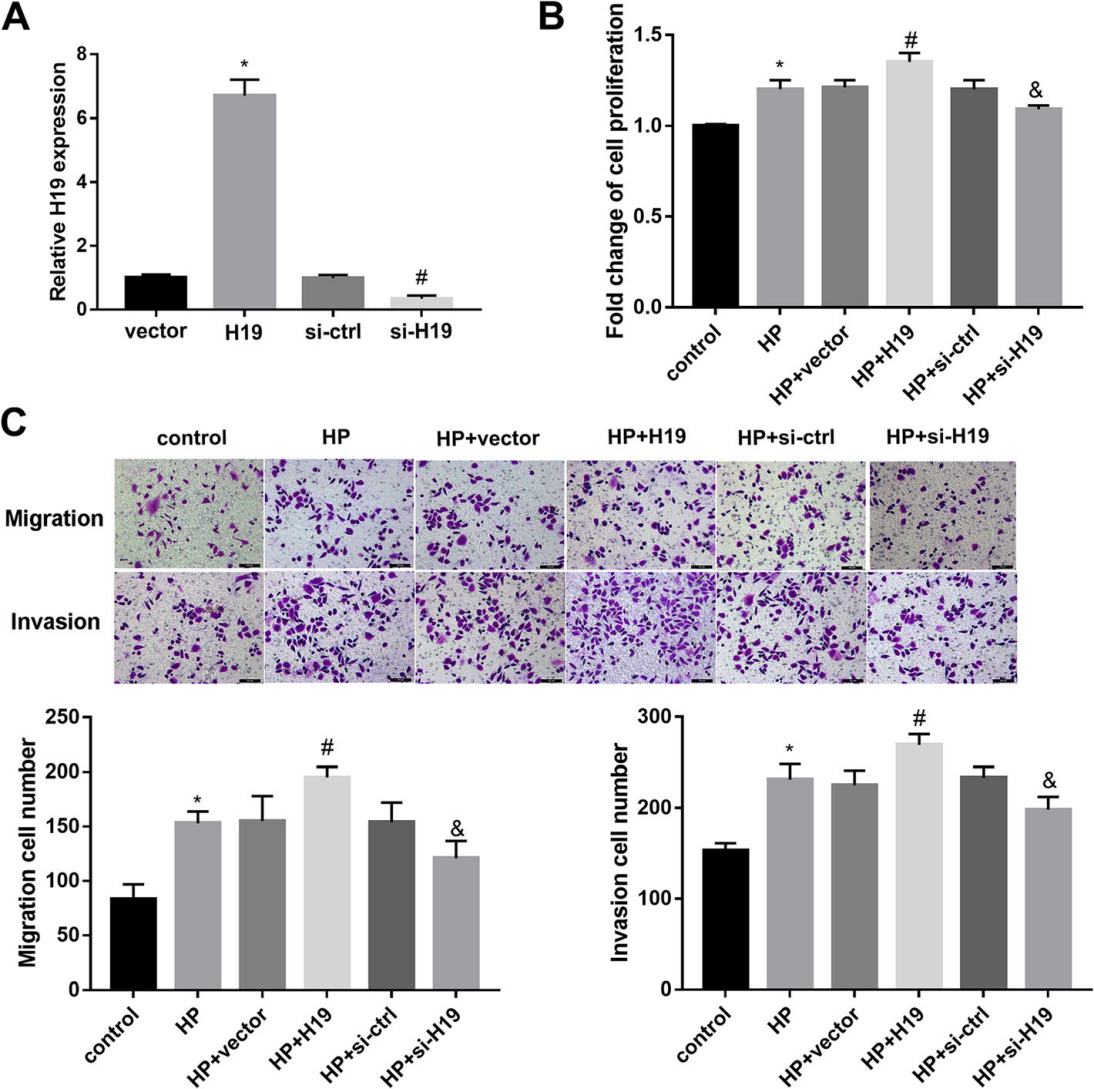
The effect of H19 on cell behaviors of SGC-7901 cells infected with HP. SGC-7901 cells were transfected with H19, si-H19 and their respective controls, followed by HP infection at MOI = 100 for 24 h. **a** The overexpression and knockdown efficiencies of H19 were confirmed by qRT-PCR. **b** Cell proliferation was analyzed using a CCK-8 kit. **c** Cell migration and invasion were analyzed using transwell assays. Each experiment was repeated three times. *p < 0.05 vs. vector or control; #*p* < 0.05 vs. si-ctrl or HP+ vector;& *p* < 0.05 vs. HP + si-ctrl

To gain further insight into the role of H19 in cell behavior of HP-infected GC cells, we transfected SGC-7901 cells with pcDNA3.1-H19 vector and si-H19, followed by HP infection. Data from qRT-PCR confirmed the overexpression and silencing efficiencies of H19 in cells (Additional file 1 Fig. 3a and Fig. 1). Importantly, H19 overexpression significantly promoted HP-induced cell proliferation. In contrast, H19 knockdown impaired the HP-induced cell proliferation (Fig. 3b). Cell migration and invasion in SGC-7901 cells showed a similar pattern (Fig. 3c). To sum up, these data manifested that H19 promoted HP-induced cell proliferation, cell migration and invasion.

### H19 promoted HP-induced GC cell proliferation, migration, and invasion via NF-κB signaling pathway

**Fig. 4:**
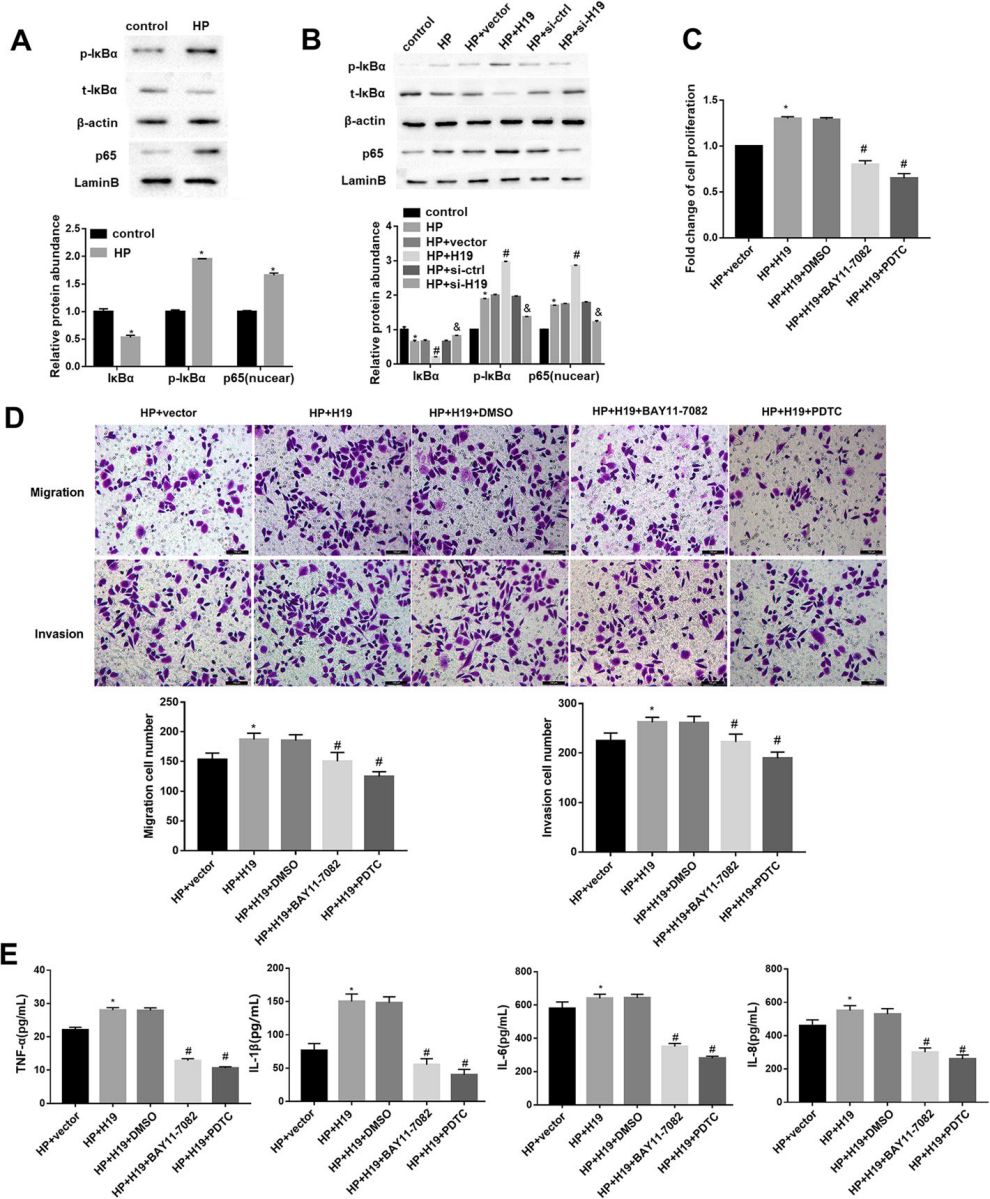
H19 promoted HP-induced GC cell proliferation, migration, and invasion via NF-κB signaling pathway. **a**: The protein levels of phosphorylated (p)-IκBα, total (t)-IκBα, and nuclear p65 in SGC-7901 cells infected with HP at MOI = 100 for 24 h were determined by western blotting. **b**: The effect of H19 on HP-activated NF-κB signaling pathway was determined by western blotting. **c**-**e**: SGC-7901 cells were transfected with H19 overexpression vector and corresponding vector controls, followed by treatment with BAY11–7082 and PDTC. After which, cells were infected with HP at MOI = 100 for 24 h. **c**: Cell proliferation was analyzed using a CCK-8 kit. **d**: Cell migration and invasion were analyzed using transwell assays. **e**: The pro-inflammatory cytokines levels were determined by ELISA. Each experiment was repeated three times. *p < 0.05 vs. control or HP + vector; #p < 0.05 vs. HP + H19+ DMSO

**Fig. 5:**
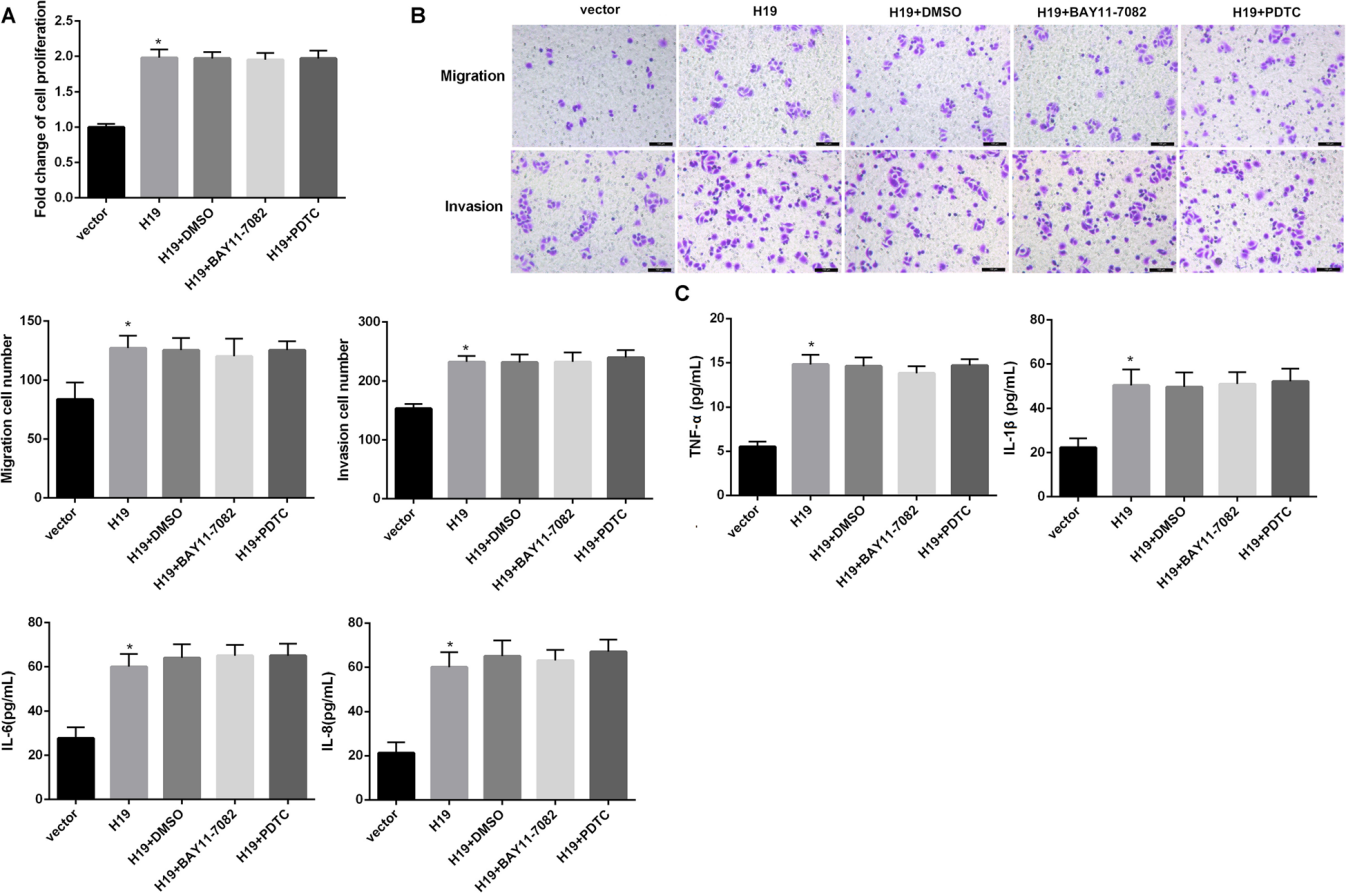
The reversal effect of NF-κB inhibitors on H19 effect was dependent on HP infection. SGC-7901 cells were transfected with H19 overexpression vector and corresponding vector controls, followed by treatment with BAY11–7082 and PDTC. **a** Cell proliferation was analyzed using a CCK-8 kit. **b** Cell migration and invasion were analyzed using transwell assays. **c** The pro-inflammatory cytokines levels were determined by ELISA. Each experiment was repeated three times. *p < 0.05 vs. vector

Finally, we sought to determine whether NF-κB signaling pathway was involved in the H19-mediated regulation of GC cell behavior. Western blotting analyses showed that HP infection significantly promoted IκBα degradation and promoted IκBα phosphorylation and nuclear p65 expression in SGC-7901 cells (Fig. 4a), indicating that HP infection activated the NF-κB signaling pathway. Importantly, the ability of HP to repress IκBα expression was markedly enhanced by H19 and compromised by si-H19, and an opposite pattern was observed for expression of p-IκBα and nuclear p65 (Fig. 4b). In addition, BAY11–7082 and PDTC (NF-κB inhibitors) abolished the H19 overexpression-mediated reduction of IκBα and elevation of p-IκBα and nuclear p65 (Supplementary Fig. 1B). Furthermore, treatment with BAY11–7082 and PDTC remarkably slowed down the HP + H19-induced cell proliferation, migration and invasion (Fig. 4c, d). Furthermore, HP + H19 markedly elevated levels of TNF-α, IL-1β, IL-6 and IL-8, but these changes were rescued by BAY11–7082 and PDTC (Fig. 4e). These results together indicated that H19 promoted cell proliferation, migration and invasion of HP-infected GC cells via enhancing NF-κB-induced inflammation. However, in SGC-7901 cells without HP infection, treatment with BAY11–7082 and PDTC had no significant effect on the H19 overexpression-mediated promotion of cell proliferation (Fig. 5a), migration and invasion (Fig. 5b) as well as levels of TNF-α, IL-1β, IL-6 and IL-8 (Fig. 5c). These results suggested that the reversal effect of NF-κB inhibitors on H19 effect was dependent on HP infection.

## Discussion

In this study, we found that H19 expression was significantly increased in HP-infected GC tissues and cells. Furthermore, we demonstrated that HP induced GC cell migration, invasion and inflammatory response. Moreover, our results identified for the first time that H19 promoted proliferation, migration and invasion of HP-induced GC cells via activating the NF-κB signaling pathway. These results increase our understanding on the role of the H19 in GC with HP infection. HP is considered to be a major risk factor of GC. Our data revealed that HP infection promoted GC cell viability, migration and invasion, and up-regulated pro-inflammatory cytokines including TNF-α, IL-1β, IL-6 and IL-8. These results are in agreement with other studies [21, 22]. These cytokines are considered important mediators of gastric pathophysiology and may play critical roles in the development of gastric inflammation and GC. It has been reported that activation of NF-κB and up-regulation of IL-8 in GC cells were suggested as the critical mechanisms responsible for HP-induced chronic inflammation and gastric carcinogenesis [23]. HP may activate NF-κB signaling through classical or alternative pathways, depending on the cell type. Activation of NF-κB by HP in gastric epithelial cells was mainly through the classical pathway, which is dependent on CagA (cytotoxin-associated gene A) and its pathogenicity island [8]. Nowadays, numerous lncRNAs have been reported to play important roles in carcinogenesis at both transcriptional and post-transcriptional levels, but their specific mechanisms of action still remain elusive [24, 25]. H19 has been identified as one of the lncRNAs involved in tumorigenesis, however, its role in tumor proliferation has been controversial for a long time. A previous study implied that H19 may function as a tumor suppressor [26]. However, other studies provided conflicting evidence as to whether H19 can promote GC and thyroid cancer [27]. In our study, H19 was expressed at higher levels in HP-infected GC tissues than in adjacent tissues, and promoted the proliferation, migration, and invasion of GC cells, which is consistent with other recent studies [28, 29]. We further demonstrated that H19 promoted the proliferation, migration and invasion of HP-infected GC cells via activating the NF-κB signaling pathway. Studies have shown that NF-κB inhibitors including BAY11–7082 and PDTC indeed directly affected GC cell growth [30–32]. However, in this study, we found that the reversal effect of NF-κB inhibitors on H19-mediated GC growth was dependent on HP infection. Our previous study showed that H19/miR-675 axis promotes GC via Fas-associated death domain protein (FADD)/caspase-8/caspase-3 signaling pathway [19]. FADD/caspase-8/caspase-3 signaling pathway was an important pathway regulating cell apoptosis whereas the NF-κB is an important mediator regulating inflammation [33]. Evidence has indicated that FADD, caspase-8-related protein (Casper), and pro-caspase-8 are parts of the tumor necrosis factor receptor type 1 (TNF-R1) -induced NFκB activation pathways, whereas activated caspase-8 can negatively regulate TNF-R1-induced NF-κB activation [34]. Further investigation was required to explore the relationship between NF-κB and FADD/caspase-8/caspase-3 signaling in the context of GC.

## Conclusions

In conclusion, the present study provides evidence that H19 promotes the proliferation, migration and invasion of HP-infected GC cells via activating the NF-κB signaling pathway. These findings might provide clues so that a better understanding of the mechanisms of GC is achieved.

## Supplementary information

**Additional file 1: Fig. S1.** The silencing efficiency of H19 in SGC-7901 cells. Relative H19 expression in SGC-7901 cells transfected with si-ctrl, siRNA1-H19, and siRNA2-H19 was examined by qRT-PCR. *p < 0.05 vs. si-Ctrl.

## Data Availability

The datasets used and/or analysed during the current study are available from the corresponding author on reasonable request.
